# Superoxide Dismutase and Pseudocatalase Increase Tolerance to Hg(II) in Thermus thermophilus HB27 by Maintaining the Reduced Bacillithiol Pool

**DOI:** 10.1128/mBio.00183-19

**Published:** 2019-04-02

**Authors:** Javiera Norambuena, Thomas E. Hanson, Tamar Barkay, Jeffrey M. Boyd

**Affiliations:** a Department of Biochemistry and Microbiology, Rutgers, the State University of New Jersey, New Brunswick, New Jersey, USA; b School of Marine Science and Policy, University of Delaware, Newark, Delaware, USA; c Delaware Biotechnology Institute, Newark, Delaware, USA; d Department of Biological Sciences, University of Delaware, Newark, Delaware, USA; CEH-Oxford; University of Missouri-Columbia; Montana State University

**Keywords:** *Thermus thermophilus*, bacillithiol, iron, mercury, pseudocatalase, reactive oxygen species, superoxide dismutase, thermophile

## Abstract

Mercury (Hg) is a widely distributed, toxic heavy metal with no known cellular role. Mercury toxicity has been linked to the production of reactive oxygen species (ROS), but Hg does not directly perform redox chemistry with oxygen. How exposure to the ionic form, Hg(II), generates ROS is unknown. Exposure of Thermus thermophilus to Hg(II) triggered ROS accumulation and increased transcription and activity of superoxide dismutase (Sod) and pseudocatalase (Pcat); however, Hg(II) inactivated Sod and Pcat. Strains lacking Sod or Pcat had increased oxidized bacillithiol (BSH) levels and were more sensitive to Hg(II) than the wild type. The Δ*bshA* Δ*sod* and Δ*bshA* Δ*pcat* double mutant strains were as sensitive to Hg(II) as the Δ*bshA* strain that lacks bacillithiol, suggesting that the increased sensitivity to Hg(II) in the Δ*sod* and Δ*pcat* mutant strains is due to a decrease of reduced BSH. Treatment of T. thermophilus with Hg(II) decreased aconitase activity and increased the intracellular concentration of free Fe, and these phenotypes were exacerbated in Δ*sod* and Δ*pcat* mutant strains. Treatment with Hg(II) also increased DNA damage. We conclude that sequestration of the redox buffering thiol BSH by Hg(II), in conjunction with direct inactivation of ROS-scavenging enzymes, impairs the ability of T. thermophilus to effectively metabolize ROS generated as a normal consequence of growth in aerobic environments.

## Abstract (precis)

Thermus thermophilus is a deep-branching thermophilic aerobe. It is a member of the *Deinococcus*-*Thermus* phylum that, together with the *Aquificae*, constitute the earliest branching aerobic bacterial lineages; therefore, this organism serves as a model for early diverged bacteria (R. K. Hartmann, J. Wolters, B. Kröger, S. Schultze, et al., Syst Appl Microbiol 11:243–249, 1989, https://doi.org/10.1016/S0723-2020(89)80020-7) whose natural heated habitat may contain mercury of geological origins (G. G. Geesey, T. Barkay, and S. King, Sci Total Environ 569-570:321–331, 2016, https://doi.org/10.1016/j.scitotenv.2016.06.080). T. thermophilus likely arose shortly after the oxidation of the biosphere 2.4 billion years ago. Studying T. thermophilus physiology provides clues about the origin and evolution of mechanisms for mercury and oxidative stress responses, the latter being critical for the survival and function of all extant aerobes.

## INTRODUCTION

All aerobes face oxidative stress, which occurs when the balance between prooxidants and antioxidants is tipped toward prooxidants. Reactive oxygen species (ROS) are prooxidants that are produced by reduction of dioxygen. This can happen intracellularly through the interaction of dioxygen with reduced flavin prosthetic groups (1). The transfer of one or two electrons to dioxygen produces superoxide (O_2_^−^) and hydrogen peroxide (H_2_O_2_), respectively (2). A three-electron transfer catalyzed by redox-active divalent transition metals, such as copper and iron (Fe) via Fenton and Haber-Weiss reactions, can produce hydroxyl radicals (^•^OH). These radicals are short-lived and rapidly react with multiple cellular constituents, including DNA (2).

Mercury (Hg) does not perform redox chemistry under biological conditions, but in animal models, Hg(II) exposure results in oxidative stress (3–7). Increased ROS upon Hg(II) exposure is thought to result from the depletion of cellular redox buffers (3, 6, 8) and/or the inhibition of the electron transport chain, allowing electrons to accumulate on flavoproteins (4, 6, 9). In bacteria, Hg(II) triggered the release of Fe(II) from solvent-exposed iron sulfur (Fe-S) clusters (10). Oxidation of solvent-accessible 4Fe-4S clusters by superoxide or H_2_O_2_ also results in Fe(II) release (11, 12). An increased pool of nonchelated or “free” cytosolic Fe(II) can accelerate Fenton chemistry (13).

Metabolic subsystems have evolved to detoxify Hg(II). Resistance to Hg(II) is encoded by the mercury resistance operon (*mer*), which is widely distributed over the bacterial and archaeal kingdoms (14). The gene composition of this operon varies among organisms, but all *mer* operons encode the mercuric reductase (MerA), which reduces Hg(II) to elemental mercury. Elemental Hg is volatile and diffuses out of the cell. Many *mer* operons also encode components involved in Hg(II) sequestration and/or transport and the Hg(II)-responsive transcriptional regulator of the operon, MerR (14). Deeply branching microbes have simple *mer* operons (15); the Thermus thermophilus operon is composed of *merA*, *merR*, and *oah2*. The latter encodes a homolog of *O*-acetyl-homocysteine sulfhydrylase, an enzyme normally involved in methionine biosynthesis and recycling (16, 17).

Our knowledge of the *mer* system comes from studies with the most derived taxa, including Escherichia coli, *Pseudomonas*, and *Bacillus*. T. thermophilus is a deep-branching thermophilic organism that responds differently to Hg(II) exposure than E. coli does (17). It possesses a different set of enzymes to detoxify ROS and uses bacillithiol (BSH) as the primary low-molecular-weight (LMW) thiol (17). T. thermophilus accumulates high concentrations of intracellular sulfides (324.1 ± 88.4 nmol/g [dry weight]) (17). Mercury has a high affinity for cellular thiols (18, 19), and exposure to 3 µM Hg(II) completely depleted free BSH pools in T. thermophilus (17). Interestingly, the cellular concentration of BSH is predicted to be an order of magnitude higher than the concentration of Hg(II) that depleted the reduced BSH pool (presented herein and in reference 17), suggesting that sequestration of BSH by Hg(II) is depleting only a portion of the BSH pool. These findings have led us to ask what happens to the rest of the BSH upon Hg(II) challenge. The disturbance of thiol-containing redox buffers, which play critical roles in ROS detoxification and oxidized protein repair, can lead to ROS accumulation (20). There is not a clearly established connection between Hg(II) and ROS in microbes, and even less is known about physiologically diverse microbes like T. thermophilus that utilize alternative redox buffers such as BSH.

We tested the overarching hypothesis that exposure of T. thermophilus to Hg(II) increases ROS accumulation because of decreased availability of reduced BSH. We demonstrate that Hg(II) exposure results in ROS accumulation. This is, in part, the result of Hg(II)-dependent inactivation of the ROS-metabolizing enzymes superoxide dismutase (Sod) and pseudocatalase (Pcat). Strains lacking ROS metabolizing enzymes contain decreased levels of reduced BSH and display increased sensitivity to Hg(II). Hg(II) exposure also inactivated aconitase, which requires a solvent-accessible Fe-S cluster, and increased free cytosolic Fe pools. This effect likely promotes ROS generation via Fenton chemistry, which we monitored by measuring DNA damage. Taken together, these findings confirm that an enzymatic capacity to detoxify ROS is important for the maintenance of a reduced intracellular thiol pool, which is necessary to mitigate Hg(II) toxicity in T. thermophilus.

## RESULTS

### Mercury exposure results in ROS accumulation and inactivates ROS-scavenging enzymes.

**FIG 1:**
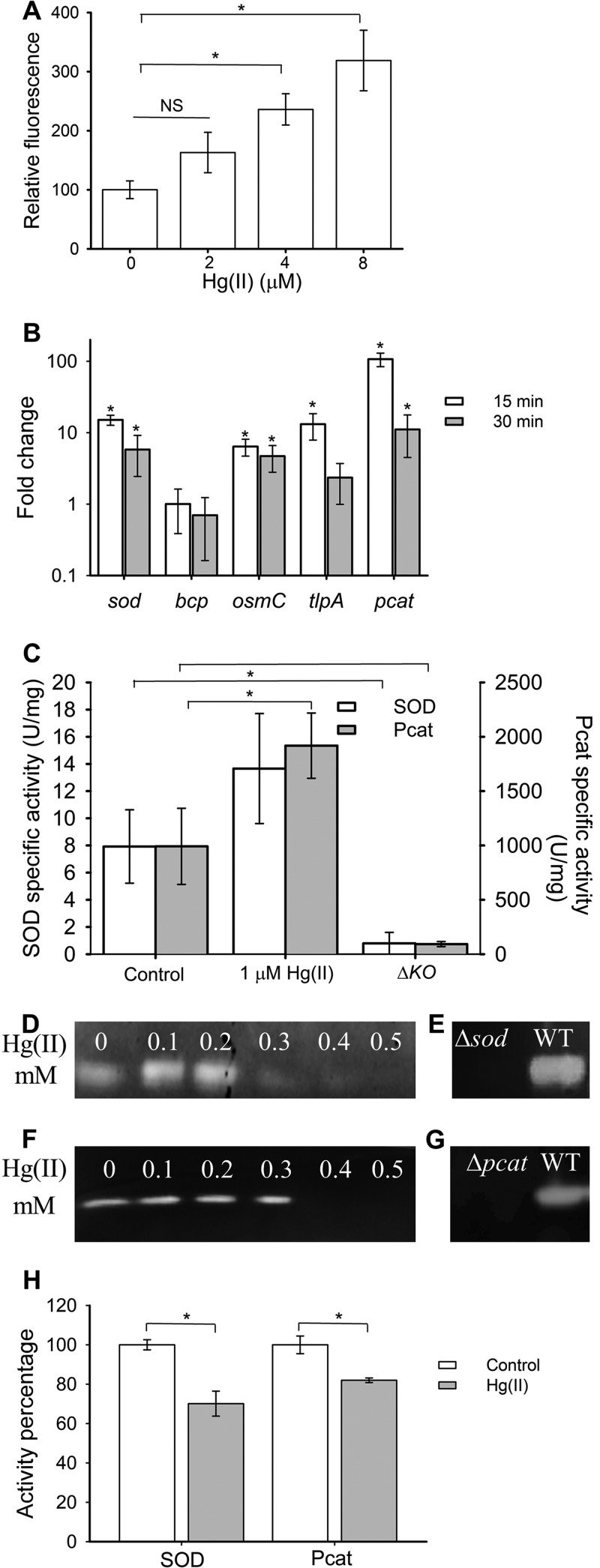
Mercury exposure induces ROS, increases Sod and Pcat expression, and inhibits SOD and Pcat activities. (A) Cultures of T. thermophilus (WT) were exposed to various concentrations of Hg(II) for 60 min before total ROS was measured using H_2_DCFDA. (B) Induction of superoxide dismutase (*sod*), bacterioferritin comigratory protein (*bcp*), organic hydroperoxide reductase (*osmC*), thiol peroxidase (*tlpA*), and pseudocatalase (*pcat*) transcription was measured in the WT strain after 15 or 30 min of exposure to 1 µM Hg(II). (C) WT cells were exposed to 0 or 1 µM Hg(II) for 30 min and superoxide (white) and H_2_O_2_ (gray) consumption was monitored. Each activity was compared to the activity of the respective Δ*sod* or Δ*pcat* mutant strain (indicated as Δ*KO* in the figure) not exposed to Hg(II). (D to G) Crude protein extracts of the WT strain were incubated with different Hg(II) concentrations. Qualitative zymograms were revealed for SOD activity (D and E) or Pcat activity (F and G). Cell extracts of the WT and Δ*sod* strains (E) or WT and Δ*pcat* strains (G) are also shown. (H) WT cells were exposed to 150 µg/ml of chloramphenicol and incubated for 30 min with 0 (white) or 5 µM Hg(II) (gray); superoxide (SOD) and H_2_O_2_ (Pcat) consumption were monitored and activity percentages (relative to the unexposed strain [control]) are shown. For panels A, B, and C, each point represents the average from at least three independent experiments and standard deviations (error bars) are shown. For panel H, three replicate experiments are shown. Student’s *t* tests were performed on the data in panels A and C, and an asterisk indicates a *P* of ≤0.05. A Mann-Whitney rank sum test was performed on the data in panel B, and an asterisk indicates a *P* of ≤0.05. NS, not significant.

We tested the hypothesis that Hg(II) exposure would increase ROS accumulation in T. thermophilus. After exposure to Hg(II), total intracellular ROS levels were qualitatively assessed with the fluorescent probe 2′,7′-dichlorodihydrofluorescein diacetate (H_2_DCFDA). Exposure to 4 or 8 µM Hg(II) for 60 min resulted in a significant increase in DCFDA-based fluorescence, suggesting increased ROS accumulation (Fig. 1A).

The genome of T. thermophilus encodes one manganese-dependent superoxide dismutase (Sod) to detoxify superoxide. It does not possess catalase, but instead, it encodes a nonheme catalase, or pseudocatalase (Pcat), that utilizes an active site manganese to metabolize H_2_O_2_ (21). It also possesses two types of peroxiredoxins: osmotically inducible protein (OsmC) and bacterioferritin comigratory protein (Bcp). These are members of the thiol peroxidase family, which catalyze the reduction of hydroperoxides (22, 23). The genome also encodes a thioredoxin-related protein, thiol:disulfide interchange protein (TlpA), which is involved in oxidative stress responses (24).

We tested the hypothesis that Hg(II) exposure increases the transcription of genes involved in ROS detoxification. Exposure of T. thermophilus to 1 µM Hg(II) increased transcript levels of *sod*, *pcat*, *osmC*, and *tlpA* (Fig. 1B). This induction was noted after 7.5 min (not shown) and sustained for at least 30 min after Hg(II) exposure (Fig. 1B). Only *bcp* transcript levels were not significantly changed. The strongest induction was observed after 15 min of Hg(II) exposure. The greatest induction was noted for *pcat*, which was induced 107 ± 23-fold.

We next examined whether the increased transcription of *sod* and *pcat* would correlate with increased enzyme activity. Cells were exposed to Hg(II) for 30 min, and then H_2_O_2_ and superoxide (SOD)-scavenging activities were measured in cell-free lysates. Hg(II) exposure significantly increased H_2_O_2_ consumption by approximately twofold. A Δ*pcat* mutant strain lost >90% of the H_2_O_2_ consumption activity, suggesting that Pcat functions in H_2_O_2_ metabolism (Fig. 1C). Superoxide consumption appeared to increase relative to the unexposed control, but it was not statistically significant (*P* = 0.249) (Fig. 1C). A Δ*sod* strain displayed eightfold-lower superoxide-scavenging activity than the Hg(II)-unexposed parent, correlating superoxide consumption with the presence of Sod.

Comparing the transcript levels and enzymatic activities revealed a significant disconnect. Hg(II) exposure resulted in greatly increased *sod* and *pcat* transcript levels, without a commensurate increase in Sod and Pcat activities. We tested the hypothesis that Hg(II) exposure was detrimental to Sod and Pcat activities. We used T. thermophilus cell-free lysates generated from cells that had not been exposed to Hg(II). Incubation of the cell-free lysate with 100 μM Hg(II) resulted in a 70% decrease in Pcat activity (see Fig. S1C in the supplemental material). We were not able to conduct traditionally described SOD assays because xanthine oxidase was inhibited by Hg(II); therefore, we measured Sod and Pcat activities by zymography. When lysates were directly exposed to Hg(II), the activities of both Sod and Pcat were decreased (Fig. 1D and F). Gel-localized activities were verified using the Δ*sod* and Δ*pcat* strains (Fig. 1E and G). The Δ*sod* and Δ*pcat* strains were more sensitive to paraquat and H_2_O_2_, respectively (Fig. S1).

10.1128/mBio.00183-19.1 FIG S1 Δ*sod*, Δ*pcat*, and Δ*bshA* strains are more sensitive to ROS than the WT strain, and Pcat is inhibited by Hg(II). (A) The WT, Δ*sod*, and Δ*bshA* strains were grown with and without paraquat, and optical densities of the cultures after 18 h of growth are shown. Growth in the unexposed control was considered 100% growth. (B) The zone of clearing monitored after exposure to 10 mM H_2_O_2_ was evaluated on soft agar plates. (C) Cell-free lysates from the WT strain were exposed to 0 to 100 µM Hg(II) before catalase activity was determined. Each point represents the average from three independent cultures, and standard deviations are shown. Student’s *t* tests were performed on the data, and an asterisk indicates a *P* of ≤0.05. Download FIG S1, TIF file, 4.6 MB. Copyright © 2019 Norambuena et al. 2019 Norambuena et al. This content is distributed under the terms of the Creative Commons Attribution 4.0 International license.

We examined whether Hg(II) affects Pcat and Sod *in vivo*. To this end, we stopped protein synthesis and incubated cells with 1 or 5 µM Hg(II) and without Hg(II) before H_2_O_2_ and superoxide consumption was monitored in cell-free lysates. In the absence of Hg(II), Pcat and Sod activities were approximately 50% after 30-min exposure to chloramphenicol (533 ± 26 and 3.9 ± 0.3 U/mg protein, respectively). Exposure to 5 µM Hg(II) further decreased consumption of superoxide by 25% (to 2.9 ± 0.3 U/mg) and consumption of H_2_O_2_ by 15% (to 453 ± 15 U/mg) compared to the cells treated with chloramphenicol only (Fig. 1H). Incubation with 1 µM Hg(II) did not result in a significant decrease in SOD or Pcat activities (not shown). These findings demonstrate that Hg(II) exposure resulted in ROS accumulation and increased activities of Pcat and Sod *in vivo*. However, the strong transcriptional induction of *pcat* and *sod* translated into only modest increases in Sod and Pcat activities. This could be, in part, the result of Hg(II) inhibition of the holoenzyme or of enzyme maturation.

### Strains lacking superoxide- or H_2_O_2_-scavenging activities are more sensitive to Hg(II).

**FIG 2:**
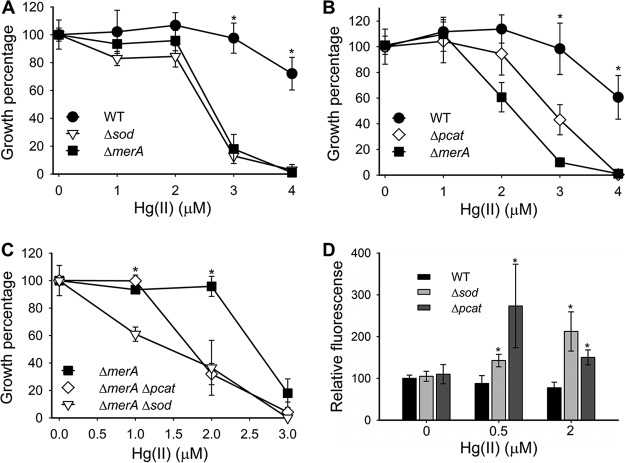
T. thermophilus strains lacking superoxide- or H_2_O_2_-scavenging activities are more sensitive to Hg(II) and have increased ROS levels upon Hg(II) exposure. The optical densities of cultures were determined after 21 h (A and C) or 18 h (B) of growth. Growth in the unexposed control was considered 100% growth. (D) Cultures were grown, and one-half of each culture was exposed to Hg(II) for 60 min before ROS were quantified using DCFDA. The fluorescence obtained for the unexposed WT strain was considered 100% fluorescence. Each point represents the average from three independent cultures, and standard deviations are shown. Student’s *t* tests were performed, and an asterisk indicates a *P* of ≤0.05.

We tested the hypothesis that Sod and Pcat have roles in mitigating Hg(II) toxicity. Compared to the parent strain (WT), T. thermophilus Δ*sod* and Δ*pcat* mutants had increased sensitivity to Hg(II) with 50% inhibitory concentrations (IC_50_) of 2.5 µM and 3 µM, respectively (Fig. 2A and B). The WT IC_50_ for Hg(II) was 4.5 µM. The Δ*sod* strain was as sensitive to Hg(II) as the Δ*merA* strain. Genetic complementation of the Δ*sod* (Fig. S2A and C) and Δ*pcat* (Fig. S2B and D) strains verified that the lack of Sod or Pcat was responsible for the observed phenotypes.

10.1128/mBio.00183-19.2 FIG S2 Genetic complementation of Δ*sod* and Δ*pcat* strains. (A) Zymogram showing superoxide consumption activity in cell lysates from the WT, Δ*sod*, and complemented Δ*sod rrsB*::*sod* (*rrsB*::*sod* in the figure) strains. (B) Zymogram showing hydrogen peroxide consumption activity of cell-free lysates from the WT, Δ*pcat*, and complemented Δ*pcat rrsB*::*pcat* (*rrsB*::*pcat* in the figure) strains. (C and D) Hg(II) resistance for WT, Δ*sod*, and *rrsB*::*sod* (*rrsB*::*sod* in the figure) strains (C) and WT, Δ*pcat*, and *rrsB*::*pcat* (*rrsB*::*pcat* in the figure) strains (D). Culture optical densities were determined after 21 h of growth. Growth in the unexposed control was considered 100% growth. Pictures of zymograms are representative of three independent experiments. Each point represents the average from three independent experiments, and standard deviations are shown. Student’s *t* tests were performed on the data, and an asterisk indicates that the value is significantly different (*P* of ≤0.01) compared to the WT value. Download FIG S2, TIF file, 5.0 MB. Copyright © 2019 Norambuena et al. 2019 Norambuena et al. This content is distributed under the terms of the Creative Commons Attribution 4.0 International license.

We tested the hypothesis that the roles of Sod or Pcat in mitigating Hg(II) toxicity were independent of the function of MerA. We compared the Hg(II) sensitivities of the Δ*merA* Δ*sod* and Δ*merA* Δ*pcat* double mutants to that of the Δ*merA* mutant. The double mutant strains were more sensitive to Hg(II) than the Δ*merA* strain (Fig. 2C), suggesting that the roles of Sod, Pcat, and MerA in Hg(II) resistance are independent and complementary.

We next tested the corollary hypothesis that ROS accumulation would occur at lower Hg(II) concentrations in the Δ*sod* and Δ*pcat* strains compared to the WT strain. We were unable to detect ROS accumulation in the Δ*sod* and Δ*pcat* strains in the absence of Hg(II) (Fig. 2D). ROS accumulation was noted in the Δ*sod* and Δ*pcat* strains upon exposure to 0.5 and 2 µM Hg(II), whereas no change in ROS levels were noted in the WT strain. These results led us to conclude that Sod and Pcat mitigate Hg(II) toxicity by controlling ROS accumulation.

### T. thermophilus strains lacking Sod or Pcat contain smaller reduced BSH pools.

**FIG 3:**
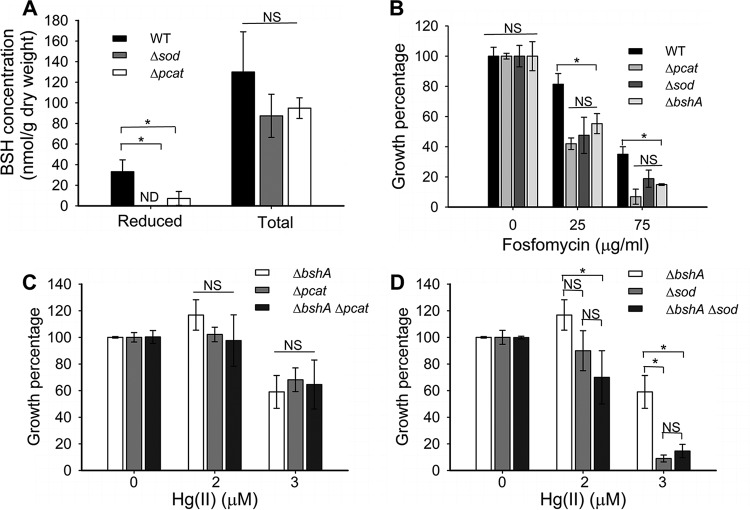
T. thermophilus strains lacking Sod or Pcat have decreased levels of reduced BSH pools. (A) Cultures were grown to an OD_600_ of 0.3 and exposed to 10 mM DTT or not exposed to DTT for 30 min before LMW thiols were quantified with mBrB. DTT-treated cells were used to measure total BSH. (B) Final culture optical densities were recorded after 20 h of growth in cultures exposed to various concentration of fosfomycin. (C and D) Effect of Hg(II) on cell growth was evaluated after 20 h of growth in the Δ*pcat*, Δ*bshA*, and Δ*pcat* Δ*bshA* strains (C) and in the Δ*sod*, Δ*bshA*, and Δ*sod* Δ*bshA* strains (D). Unexposed controls were considered 100% growth. Each point represents the average from three independent cultures, and standard deviations are shown. Student’s *t* tests were performed on the data, and an asterisk indicates a *P* of ≤0.05. NS, not significant; ND, no signal detected.

We tested the hypothesis that BSH functions in metabolizing ROS or the by-products of ROS damage. We quantified the reduced BSH pools in the Δ*sod* and Δ*pcat* strains by monobromobimane derivatization and HPLC, which quantifies free BSH pools. Free BSH was not detected in the Δ*sod* mutant (Fig. 3A), and the Δ*pcat* strain had 80% less free BSH than the WT strain (7.2 ± 6.7 versus 33.2 ± 10.2 nmol g^−1 ^[dry weight] for the WT) (Fig. 3A). Importantly, all strains had approximately the same intracellular concentration of total (reduced plus oxidized) BSH (Fig. 3A), strongly suggesting that the lack of free BSH is due to its oxidation in the mutant strains or defective recycling of bacillithiol disulfide (BSSB) back to BSH. The same HPLC traces did not display a significant difference in intracellular sulfide concentrations between the WT, Δ*sod*, and Δ*pcat* strains, but these peaks were quite broad, making it difficult to quantify accurately (data not shown).

Reduced BSH is required to detoxify the antibiotic fosfomycin (25) and mitigate oxidative stress (26). The Δ*sod* and Δ*pcat* strains were more sensitive to fosfomycin than the WT and had fosfomycin sensitivities similar to that of the Δ*bshA* strain (Fig. 3B), which cannot synthesize BSH (17). Compared to the WT strain, the Δ*bshA* strain showed increased sensitivity to H_2_O_2_ and paraquat; however, the Δ*bshA* strain was less sensitive to the toxicants than the Δ*sod* and Δ*pcat* strains (Fig. S1).

ROS-scavenging deficient strains were constructed in the Δ*bshA* background to test whether Hg(II) sensitivity in the Δ*pcat* and Δ*sod* strains was exacerbated by a complete lack of BSH (17). The Hg(II) sensitivity phenotypes corresponding to the Δ*bshA* and Δ*pcat* mutations were not additive (Fig. 3C), but the Δ*sod* strain was more sensitive to 3 µM Hg(II) than the Δ*bshA* strain was (Fig. 3D). These results suggested that Sod has a role in preventing Hg(II) toxicity in addition to its role in preventing the oxidation of the BSH pool, while the Hg(II) sensitivity of the Δ*pcat* strain appears to result from a lack of reduced BSH.

### Hg(II) exposure results in decreased aconitase (AcnA) activity, increased free cytosolic Fe, and DNA damage.

**FIG 4:**
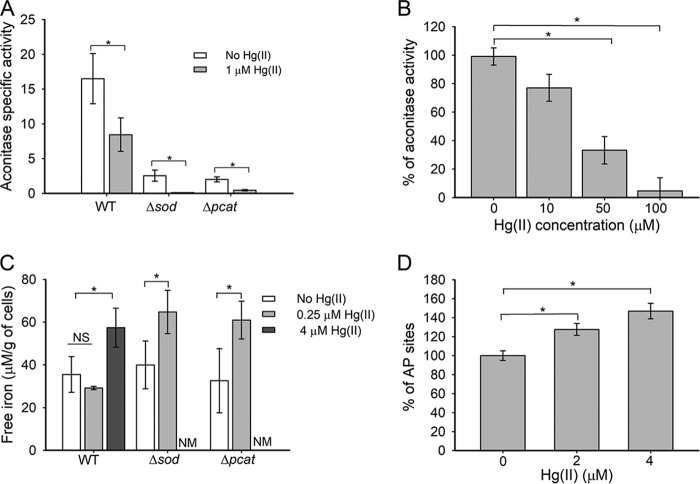
Hg(II) stress results in aconitase inactivation, increased intracellular free iron, and DNA damage. (A) Aconitase activity was monitored in cell-free lysates after whole cells had been exposed to 1 µM Hg(II) or not exposed to Hg(II) for 30 min. (B) Cell-free lysates from the WT strain were exposed to 0 to 100 µM Hg(II) before aconitase activity was determined. (C) The concentration of free Fe was quantified after exposure to 0.25 µM Hg(II) or to 4 µM Hg(II) for 30 min. Cell weight is reported as wet weight. (D) DNA damage was determined by quantifying the number of apurinic/apyrimidinic sites (AP sites) in the WT strain [cells unexposed to Hg(II) had an average of 8.38 ± 0.77 AP sites per 100,000 bp of DNA]. Each point represents the average from at least three independent cultures, and standard deviations are shown. Where shown, Student’s *t* tests were conducted on the data, and an asterisk indicates a *P* of ≤0.05. NS, not significant; NM, not measured.

BSH plays a fundamental role in Hg(II) resistance in T. thermophilus, and exposure to 3 µM Hg(II) completely depleted free BSH pools (17). The Δ*sod* and Δ*pcat* strains had decreased concentrations of reduced BSH (Fig. 3A), suggesting that there may be more free Hg(II) in the cytoplasms of the Δ*sod* and Δ*pcat* strains when challenged with Hg(II). Prior work in E. coli found that Hg(II) inactivated fumarase, which requires a solvent-accessible Fe-S cluster for catalysis (10). When T. thermophilus was exposed to 1 µM Hg(II) for 30 min, AcnA activity decreased to 50% of the unexposed control (Fig. 4A). The nonchallenged Δ*pcat* and Δ*sod* strains had 12 and 16% of the activity of the WT strain, respectively (Fig. 4A). Upon exposure to Hg(II), AcnA activity was reduced a further 30-fold in the Δ*sod* strain and 4.5-fold in the Δ*pcat* strain (Fig. 4A). We next examined whether Hg(II) inactivated T. thermophilus AcnA *in vitro*. To this end, we added Hg(II) to anaerobic cell-free lysates prior to measuring AcnA activity. AcnA activity decreased as a function of Hg(II) added and was nearly undetected after exposure to 100 µM Hg(II) (Fig. 4B).

We next tested the hypothesis that Hg(II) exposure would increase the size of the cytosolic free Fe pool. T. thermophilus was exposed to 4 µM Hg(II) or not exposed to Hg(II) for 30 min, and intracellular free Fe was quantified using electron paramagnetic resonance (EPR) spectroscopy (19, 27). Exposure significantly increased the pool of free Fe by 1.7-fold (Fig. 4C). When the WT, Δ*sod*, and Δ*pcat* strains were exposed to 0.25 µM Hg(II), the WT free Fe pool was unaltered, while it was significantly increased, 1.8-fold, in the Δ*sod* and Δ*pcat* strains; however, at 4 µM Hg(II), the free Fe pool was elevated in the WT strain (Fig. 4C). Thus, treatment with a lower concentration of Hg(II) was capable of disrupting the Fe homeostasis in the Δ*sod* and Δ*pcat* strains compared to the WT. These strains had similar free Fe levels when cultured in the absence of Hg(II) (Fig. 4C).

Free Fe(II) can catalyze Fenton chemistry to produce HO^•^ (2) that can damage DNA (28) by producing apurinic/apyrimidinic (AP) sites (29, 30). We hypothesized that Hg(II) exposure would result in increased DNA damage. After exposure to either 2 or 4 µM Hg(II), there was a significant increase in AP sites (Fig. 4D). Repair of AP sites requires base excision repair, which in T. thermophilus depends on the Nfo endonuclease IV (31). A T. thermophilus Δ*nfo* mutant was more sensitive to Hg(II) than the WT strain (Fig. S3).

10.1128/mBio.00183-19.3 FIG S3 A Δ*nfo* strain is more sensitive to Hg(II) than the WT. Strains were cultured with various concentrations of Hg(II), and final optical densities were measured after 20 hours. Growth in the unexposed control was considered 100% growth. Each point represents the average from at least three independent cultures, and bars represent standard deviations. Student’s *t* tests were performed against the WT strain, and an asterisk indicates a *P* of ≤0.001. Download FIG S3, TIF file, 2.0 MB. Copyright © 2019 Norambuena et al. 2019 Norambuena et al. This content is distributed under the terms of the Creative Commons Attribution 4.0 International license.

Taken together, these data are consistent with a model wherein Hg(II) exposure decreases the activities of enzymes requiring solvent-exposed Fe-S clusters and increases intracellular free Fe. The increase in free Fe likely contributes to increased hydroxyl radicals resulting in increased DNA damage.

## DISCUSSION

The mechanisms by which metals exert toxicity are not fully understood. These phenomena have largely been examined in model organisms, and relatively few studies have been conducted in physiologically or phylogenetically diverse organisms. In this study, we examined the effect of Hg(II) exposure on a deeply branching thermophilic bacterium to expand our knowledge of Hg(II) toxicity and tolerance in phylogenetically and physiologically diverse microbes.

**FIG 5:**
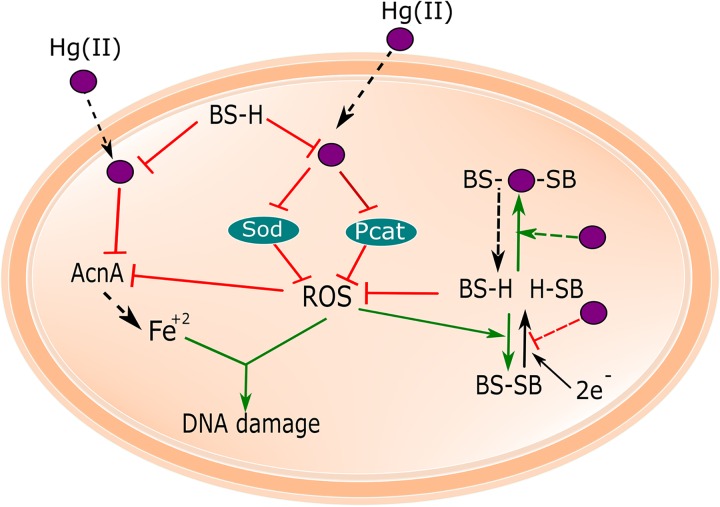
Working model for ROS generation by Hg(II). Exposure of T. thermophilus to Hg(II) (purple) results in the inactivation of two ROS-detoxifying enzymes (Sod and Pcat) and ROS accumulation. Hg(II) decreases bioavailable BSH, which is necessary to prevent Hg(II) intoxication and ROS accumulation. The presence of Sod and Pcat are necessary to maintain reduced BSH pools, as well as to metabolize superoxide and H_2_O_2_, respectively. Hg(II) accumulation inactivates enzymes, such as aconitase, with solvent-accessible Fe-S clusters and increases intracellular free Fe. The free Fe^2+^ participates in Fenton chemistry producing hydroxyl radicals, which damage DNA. Systems inhibited are shown in red, and systems favored upon Hg(II) toxicity are shown in green.

Data presented herein, and from our previous study (17), have led to a working model for how Hg(II) exposure affects T. thermophilus (Fig. 5). In our model, increased titers of cytosolic Hg(II) result in ROS accumulation, which also may be the result of Hg(II)-dependent inactivation of Sod and Pcat. Strains lacking Sod or Pcat have increased levels of oxidized BSH. Reduced BSH is necessary to buffer both cytosolic Hg(II) and ROS. In the absence of reduced BSH, Hg(II) accumulation inactivates enzymes, such as aconitase, with solvent-accessible Fe-S clusters and increases intracellular free Fe. The increased free Fe(II) participates in Fenton chemistry, resulting in an increase in hydroxyl radicals causing DNA damage. Thus, exposure to Hg(II) results in oxidative stress even though Hg(II) is not a redox-active metal, and mutations that diminish cellular defenses against ROS indirectly increase Hg(II) sensitivity. It is also probable that BSH directly acts as a Hg(II) ligand (17, 32).

Oxidative stress among the prokaryotes has been mostly examined in E. coli with little attention to physiologically diverse microbes. *Thermus* spp. inhabit hot environments where heat lowers maximal oxygen saturation (4.53 mg/liter at 65°C) relative to saturation under conditions utilized to culture E. coli (6.73 mg/liter at 37°C) (33). When tested, Thermus aquaticus grew better under microaerophilic conditions compared to more aerated conditions, correlating with a decreased ability to detoxify ROS (34). These facts may also explain the presence of pseudocatalase rather than catalase (35). Relative to E. coli, T. thermophilus displays a distinct gene expression pattern upon Hg(II) exposure. In T. thermophilus, *sod*, *pcat*, *osmC*, and *tlpA* transcripts, but not *bcp*, were induced in response to Hg(II) (Fig. 1A). In E. coli, Hg(II) was found to induce the expression of *sodB* and the peroxiredoxin *ahpC*, but not *katG* and *katE* that encode catalases (36). The E. coli *sodA*, which is the T. thermophilus *sod* orthologue, was repressed by short-term Hg(II) exposure (36). We previously showed that E. coli and T. thermophilus differentially regulate the transcription of genes required for LMW-thiol synthesis upon Hg(II) exposure (17). Taken together, these findings highlight the fact that these two bacteria, one deep branching and the other highly derived, differ in their responses to Hg(II). The findings reported here, therefore, provide a foundation for future studies to decipher how microbial systems have evolved in response to the combined toxic effects of metals and oxygen.

The amount of BSH in T. thermophilus cells appears to be lower than the concentration of glutathione typically found in Gram-negative bacteria. Assuming that T. thermophilus cells have the same volume and dry weight as E. coli cells, the cytosolic concentration of BSH would be ∼40 μM under the growth conditions utilized. Previous work found the concentration of BSH in Bacillus subtilis and Deinococcus radiodurans to be ∼200 μM (37). The concentration of glutathione in E. coli cells is ∼5 mM (37). The lower concentration of BSH in T. thermophilus cells could constrain the ability to use BSH to buffer against ROS when Hg(II) accumulates in the cytosol. This could result in an increased reliance on alternative ROS-mitigating factors, such as Sod, to protect the cell.

BSH pools were decreased by incubation with Hg(II) (17) and in mutant strains lacking Pcat or Sod. We found that BSH functions to prevent ROS poisoning in T. thermophilus (see Fig. S1 in the supplemental material). The Δ*sod* and Δ*pcat* strains had lower levels of reduced BSH, but the same overall concentration of BSH (Fig. 3A), suggesting that ROS or a by-product of ROS metabolism results in increased BSH oxidation. A role for BSH as a buffer against ROS accumulation could explain why there was no detected difference in ROS titers in the Δ*sod*, Δ*pcat*, and WT strains in the absence of Hg(II). It is currently unknown which enzyme(s) is (are) responsible for reducing BSSB back to BSH in T. thermophilus. In yeast and protists, glutathione reductase is inhibited by Hg(II) (38, 39) and if T. thermophilus utilizes a similar enzyme to reduce BSSB, which is likely, it is possible that this enzyme is also inhibited by Hg(II), resulting in a decreased ability to recycle BSSB to BSH (Fig. 5). It was hypothesized that YpdA functions as a BSSB reductase in B. subtilis (40). The genome of T. thermophilus encodes a gene product that is 39% identical to YpdA (NCBI accession no. YP_144481). Future studies will be necessary to determine the effect of this gene product on BSSB recycling.

In some cyanobacteria, glutaredoxin reductase possesses a mercuric reductase activity (41), and it is thus conceivable that MerA in *Thermus* may serve as a BSSB reductase. This possibility is hard to evaluate with our current mechanistic understanding of MerA, which is largely based on studies with proteobacterial reductases (42, 43). MerA in *Thermus* is a core MerA, lacking the 70-amino-acid N terminus (NmerA) (44) that functions in delivering S-Hg-S to the redox active site of the enzyme (43) and thus must differ from the full-length proteobacterial variants in interaction with its substrates. We also found that the reduced BSH pool in the Δ*merA* strain was similar to that of the WT (not shown).

We previously reported the high concentrations of sulfides in strain HB27 (324.1 ± 88.4 nmol/g [dry weight]) (17). The natural habitats for *Thermus* spp. are usually moderate- to high-temperature terrestrial springs with low sulfide and circumneutral to alkaline pH, suggesting a chemoorganotrophic metabolism (45, 46). However, genome sequences of several *Thermus* spp., including HB27, revealed presence of genes related to the SOX and PSR systems (47). These systems may specify mixotrophic growth with reduced sulfur as an energy source and anaerobic polysulfide respiration, respectively (48). We are not aware of reports demonstrating such metabolic capabilities in *Thermus*, and our findings in this paper and our previous paper (17) highlight the need for further research on this topic.

Hg(II) readily reacts with sulfide to form HgS, and evidence suggests that sulfide production could be a Hg(II) detoxification mechanism (49). We did not notice a significant decrease in the size of the sulfide pool upon challenge with Hg(II) (17); however, the small amount of Hg(II) added to T. thermophilus cultures relative to the size of the sulfide pool likely render it impossible to detect a decline in sulfide concentration upon Hg(II) binding. Hydrogen sulfide has been found to aid in the detoxification of ROS (50–52). In the future, we would like to decrease the size of the sulfide pool and examine the consequences on ROS metabolism and Hg(II) challenge.

Hg(II) inhibited Sod, Pcat, and AcnA *in vivo* and *in vitro*, but a higher concentration of Hg(II) was required to inhibit these enzymes *in vitro*. Moreover, the concentrations of Hg(II) necessary to inhibit SOD and Pcat *in vitro* were much higher than predicted to accumulate inside cells under the growth conditions utilized. Among the scenarios that could explain this discrepancy, the most plausible explanation might be the difference in available Hg(II) *in vivo* and *in vitro.* Mercury bioavailability is greatly affected by the presence of ligands (53–55). If cell lysis during preparation of crude cell extracts releases ligands that are compartmentalized within intact cells, these may greatly reduce Hg(II) bioavailability in *in vitro* assays. This is suggested by our laboratory’s protocols for mercuric reductase assays whereby resting cells and crude extract activities are measured at 10 and 100 µM Hg(II) (56), respectively. The high concentrations of sulfide in strain HB27 (17), which are likely present as labile organic and inorganic persulfides and polysulfides (57), may greatly limit Hg(II) bioavailability in crude cell extracts. The precise nature of the intracellular sulfide pool in strain HB27 and how it interacts with metals and other stressors will be an important future avenue of investigation.

This study reports on the effects of Hg(II) on T. thermophilus, which belongs to one of the earliest aerobic bacterial lineages (68) inhabiting high mercury environments (69). We report that ROS detoxification is important for Hg(II) tolerance; therefore, in T. thermophilus, resistance to Hg(II) is achieved through both *mer*-based detoxification (16, 17) and the oxidative stress response. We previously suggested that the *mer* system evolved in response to the oxygenation of earth due to the increased availability of oxidized Hg species (44). It is likely that these same environmental changes led to the evolution of the oxidative stress response. While numerous reports have documented metal-induced oxidative stress (reviewed in references 8, 58, and 59), few examined how responses to this stress alleviate metal toxicity among prokaryotes. Our findings in T. thermophilus alert us to these hitherto little-studied aspects of metal homeostasis.

## MATERIALS AND METHODS

### Chemicals and bacterial growth conditions.

Thermus thermophilus HB27 (WT) and its mutants were cultured at 65°C in 461 Castenholz TYE medium (complex medium [CM]) (16). When cultured in liquid medium, cells were grown in 3 ml of medium in 10-ml test tubes incubated perpendicularly and shaken at 200 rpm. Test tubes were used to grow cells for ROS analysis, RNA extraction, resistance assays, and AP site quantification. Flasks (2:3 gaseous headspace to liquid medium ratio) were used to grow cultures to generate cell extracts for enzyme assays, zymograms, thiol content determination, and for intracellular Fe concentration determination. Solid culture medium was supplemented with 1.5% (wt/vol) agar. Kanamycin (Kan) and hygromycin B were supplemented at 25 µg ml^−1^ and 40 µg ml^−1^, respectively. Unless otherwise stated, overnight (ON) cultures of T. thermophilus were diluted in fresh medium to an optical density at 600 nm (OD_600_) of 0.1 and further grown to OD_600_ of ∼0.3 before challenged with toxicants (fosfomycin, paraquat, or HgCl_2_). Mercury was used as HgCl_2_ for all assays. Protein concentrations were determined using the Quick Start Bradford protein assay (Bio-Rad Laboratories Inc., Hercules, CA).

### Mutant construction.

The in-frame deletions for *sod* (WP_011172643.1) and *pcat* (WP_011174225.1) were performed as previously described (17). DNA primers used in this study are listed in Table S1 in the supplemental material. Gene replacements were confirmed by DNA sequencing. For genetic complementation, the 16S rRNA gene (*rrsB*; TT_C3024), was replaced with the complementing gene constructs by the method of Gregory and Dahlberg (60). All mutant strains used the native gene promoter to express resistance cassettes or genes.

10.1128/mBio.00183-19.4 TABLE S1 Primers and conditions used for qPCR. Download Table S1, DOCX file, 0.01 MB. Copyright © 2019 Norambuena et al. 2019 Norambuena et al. This content is distributed under the terms of the Creative Commons Attribution 4.0 International license.

### Monitoring reactive oxygen species.

The fluorophore 2′,7′-dichlorodihydrofluorescein diacetate (H_2_DCFDA) (61–63) was used for ROS monitoring. Cells were incubated for 60 min in the presence or absence of Hg(II). Cells from 1 ml of culture were pelleted, washed with phosphate-buffered saline (PBS), resuspended in 500 µl of 10 µM H_2_DCFDA in PBS, and incubated for 30 min at 37°C. After incubation, cells were washed with PBS and lysed by sonication. Fluorescence was measured (Perkin Elmer HTS 7000 Plus Bio assay reader) at 485 nm as the excitation wavelength and 535 nm as the emission wavelength. Data were normalized to protein concentration.

### RNA extraction, cDNA synthesis, and qPCR.

For induction of gene expression, cells were exposed to 1 µM Hg(II) for 15 or 30 min. Three-milliliter aliquots were removed and mixed with RNA protect (Qiagen). RNA extraction and cDNA synthesis were performed as previously described (17). Transcripts were quantified by qPCR (iCycler iQ; Bio-Rad Laboratories Inc., Hercules, CA) as previously described (17). DNA primers and cycling temperatures used are listed in Table S2.

10.1128/mBio.00183-19.5 TABLE S2 PCR primers used to construct mutant strains. Download Table S2, DOCX file, 0.01 MB. Copyright © 2019 Norambuena et al. 2019 Norambuena et al. This content is distributed under the terms of the Creative Commons Attribution 4.0 International license.

### Enzymatic assays.

Cultures (25 ml) were exposed to Hg(II) for 30 min, cells were pelleted and washed with PBS, and cell pellets were frozen until further use. Crude cell extracts were prepared as previously described (56). All enzyme assays were performed at 50°C. For exposure of crude cell lysates, Hg(II) was added at the indicated concentrations and incubated for 5 min before measuring enzymatic activity. The assay described by Oberley and Spitz (64) was used to determine SOD activity with 30 µg of crude extract. One unit was defined as the amount of enzyme needed to reduce the reference rate by 50% (64). Measurements were carried out with an AVIV 14 UV-VI spectrophotometer. Catalase activity was measured by the method of Beers and Seizer (65) with 0.6 mg of protein extract. One unit was defined as the amount of enzyme needed to degrade 1 µmol of H_2_O_2_ per min (ε = 43.6 M^−1^ cm^−1^ for H_2_O_2_). For aconitase activity, cell lysis was performed under anaerobic conditions as described elsewhere (66) with 20 µg of protein extract. One unit was defined as the amount of enzyme needed to degrade one µmol of DL-isocitrate per s (ε = 3.6 mM^−1^ cm^−1^ for *cis*-aconitate). To determine the *in vivo* Hg(II)-dependent inhibition of H_2_O_2_ and superoxide consumptions, protein synthesis was stopped by adding 150 µg chloramphenicol/ml to cells grown to an OD_600_ of ∼0.3, before 5 µM Hg(II) was added. Cells were incubated for 30 min before harvesting as described above. Catalase and aconitase activities were measured with a UVmini-1240 spectrophotometer (Shimadzu Corporation, Kyoto, Japan).

### Resistance assays.

Overnight cultures were diluted to an OD_600_ of 0.1 in fresh CM, and various concentrations of toxicant (fosfomycin, paraquat, or HgCl_2_) were added to individual samples at different concentration ranges. Resistance was assessed as the percentage of growth observed at the indicated times relative to the control that was not exposed to the toxicant (100% growth). Soft agar assays were used to assess H_2_O_2_ sensitivity. Cells were grown as for liquid assays, and 40 µl of the culture was added to 4 ml of CM soft agar (0.8% [wt/vol]) and then poured over a 25-mm petri dish with CM agar. Ten microliters of 10 mM H_2_O_2_ was added to the center of the plate. The halo of growth inhibition was measured after 24-h incubation.

### Zymograms.

SOD and catalase in-gel activities were performed as described elsewhere (67). For SOD and Pcat activities, 25 µg and 50 µg, respectively, of cell lysates were loaded on the gels. Cell lysates were prepared as described above for enzymatic assays.

### Thiol concentration determination.

Extraction and quantification of low-molecular-weight thiols were performed as previously described (17). Briefly cells were resuspended in D-mix (acetonitrile, HEPES, EDTA, and mBrB) and incubated for 15 min at 60°C in the dark. Free thiols are complexed with mBrB before the reaction was stopped with methanesulfonic acid. Samples were centrifuged, and cell debris was separated from the soluble thiols before quantifying LMW thiols by HPLC. Cell debris was dried to determine the dry weight of cell material derived from each sample. For total BSH determinations, cells were exposed to 10 mM DTT for 30 min prior to thiol extraction.

### Intracellular iron quantification.

The intracellular iron quantification assay was performed by the method of LaVoie et al. (19). Cultures (100 ml) were exposed to Hg(II) for 30 min. Cells were pelleted by centrifugation, resuspended in 5 ml of PBS with 10 mM diethylene triamine pentaacetic acid (DTPA) and 20 mM deferoxamine mesylate salt (DF), shaken at 37°C for 15 min at 180 rpm, and pelleted at 4°C. Cells were washed once with ice-cold 20 mM Tris-HCl (pH 7.4), resuspended in the same buffer with 15% (vol/vol) glycerol, and stored at −80°C. For EPR analysis, cell suspensions were thawed on ice, and 200-μl aliquots were dispensed into 4-mm OD quartz EPR tubes and frozen in liquid nitrogen. Continuous-wave (CW) EPR experiments were performed with an X-band Bruker EPR spectrometer (Elexsys580) equipped with an Oxford helium flow cryostat (ESR900) and an Oxford temperature controller (ITC503). EPR parameters used in our experiments were as follows: microwave frequency, 9.474 GHz; microwave power, 20 mW; modulation amplitude, 2 mT; and sample temperature, 25°K. The Fe(III)/DF concentration of each sample was determined by comparing the peak-to-trough height of EPR signal at *g* = 4.3 against the standard sample with a known Fe(III)/DF concentration (50 μM FeCl_3_ and 20 mM DF in 20 mM Tris-HCl at pH 7.4 with 15% [vol/vol] glycerol).

### Quantification of apurinic or apyrimidinic (AP) sites.

Cells were exposed to Hg(II) for 60 min. Three-milliliter aliquots of cultures were pelleted and washed with PBS prior to DNA extraction using QIAamp DNA kit (Qiagen). AP sites were quantified using the Oxiselect oxidative DNA damage quantification kit (Cell Biolabs).

### Statistical analysis.

One-way ANOVA followed by a Dunnet test analysis was performed for multiple group comparison to a control. For two group comparisons (controls versus treatment), Student’s *t* tests were performed.

### Data availability.

All data will be provided upon request.
