# Supplementary figures and images for: Superoxide Dismutase and Pseudocatalase Increase Tolerance to Hg(II) in Thermus thermophilus HB27 by Maintaining the Reduced Bacillithiol Pool
Source: mBio. 2019 Apr 2;10(2):e00183-19. doi: 10.1128/mBio.00183-19 (PMC6445937; doi:10.1128/mBio.00183-19)

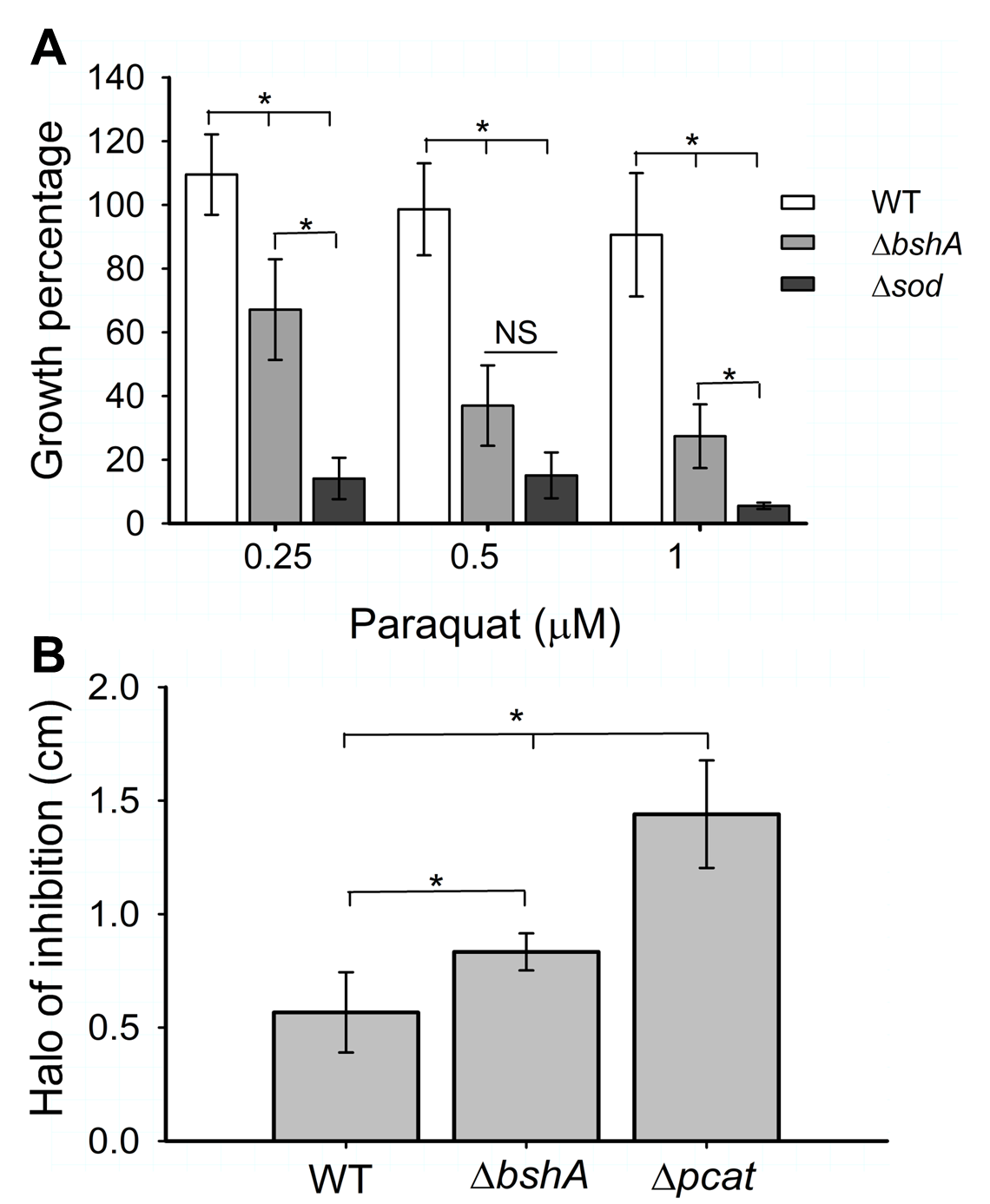

Supplement: FIG S1 [file mBio.00183-19-sf001.tif]

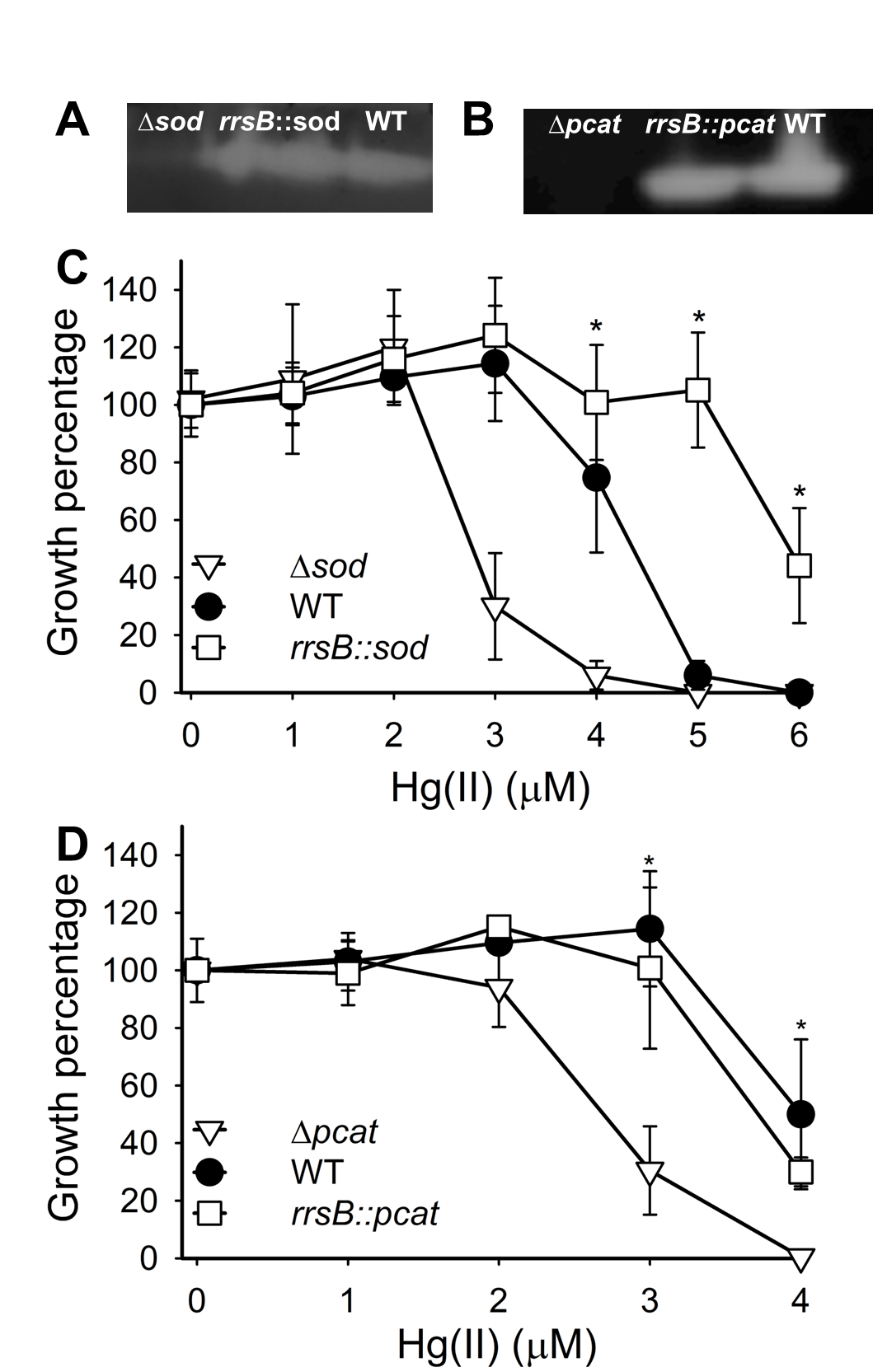

Supplement: FIG S2 [file mBio.00183-19-sf002.tif]

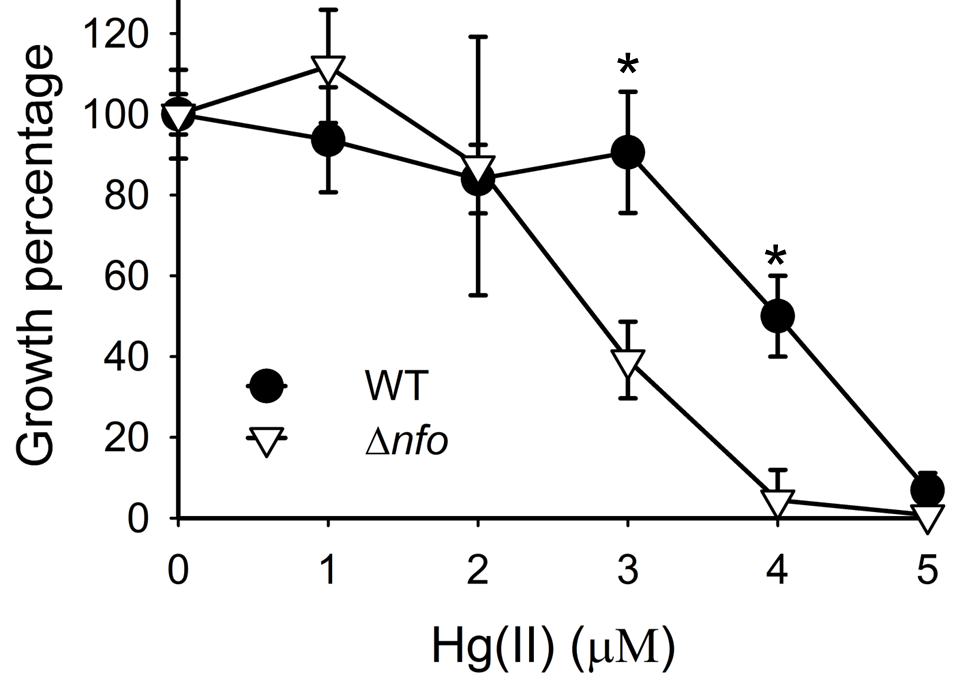

Supplement: FIG S3 [file mBio.00183-19-sf003.tif]
