# Supplementary material for: Superoxide Dismutase and Pseudocatalase Increase Tolerance to Hg(II) in Thermus thermophilus HB27 by Maintaining the Reduced Bacillithiol Pool
Source: mBio. 2019 Apr 2;10(2):e00183-19. doi: 10.1128/mBio.00183-19 (PMC6445937; doi:10.1128/mBio.00183-19)
Supplement: TABLE S1 [file mBio.00183-19-st001.docx]

**Table S1** Primers and conditions used for qPCR.

| Primer | Sequence | C* (µM) | T (°C) | Size (bp) | Source |
| --- | --- | --- | --- | --- | --- |
| gyrase-F | GGGCGAGGTCATGGGC | 1 | 61 | 134 | Norambuena et al 2018 |
| gyrase-R | CGCCGTCTATGGAGCCG | 0.25 |  |  |  |
| SOD-F | CGTTCAAGCTTCCTGACCTAGG | 1.25 | 59 | 117 | this study |
| SOD-R | CGTTGAGGTTCGTCACGTAGGC | 1.25 |  |  |  |
| osmC-F | GATTGAGCTTCTGACCGAGGC | 1.25 | 60 | 126 | this study |
| osmC-R | AGGACGATCTCCTTCACCCC | 1.25 |  |  |  |
| bcp-F | GAAGTACGGCCTGAACTTTCC | 1.25 | 58 | 132 | this study |
| bcp-R | TCTATGAGGAAGGTCTGGCG | 1.25 |  |  |  |
| TplA-F | TGGCTTTGTCTTGGAGAACGC | 1.25 | 60 | 141 | this study |
| TplA-R | CAGAGGTGTTTGGGCAAGGC | 1.25 |  |  |  |
| pcat rev | CGCCACCAGCTCAATGT | 1.25 | 57 | 105 | this study |
| pcat for | ATGTACCAGTCCTTCAACTTCC | 1.25 |  |  |  |

*C indicates final concentration of the primers
