# Supplementary material for: Superoxide Dismutase and Pseudocatalase Increase Tolerance to Hg(II) in Thermus thermophilus HB27 by Maintaining the Reduced Bacillithiol Pool
Source: mBio. 2019 Apr 2;10(2):e00183-19. doi: 10.1128/mBio.00183-19 (PMC6445937; doi:10.1128/mBio.00183-19)
Supplement: TABLE S2 [file mBio.00183-19-st002.docx]

**Table S2** PCR Primers used to construct mutant strains.

| KO strain | Primer | Sequence |
| --- | --- | --- |
| ∆*sod* | 1 fum ecoRi for | TCGCGGGAATTCAGGGGAAC |
|  | E HTK SOD rev | ATTGGTCCTTTCATACTTCACCTCCGC |
|  | A sod HTK for | GGAAGCGGAGGTGAAGTATGAAAGGACCAATAATAA |
|  | B sod htk rev | GCTATAAGGCTATGGGGATCAAAATGGTATGCGTT |
|  | 4 sod bamh1 rev | GGCGGATCCGGGCCTTA |
|  | F HTK SOD for | GCATACCATTTTGATCCCCATAGCCTTATAGC |
|  | 5 fum upins for | GGAAGGTCAACCCCACCCAG |
|  | 6 sod downins rev | AAGGCCCTCCTCTTCGGC |
| ∆*pcat* | A cat | AAAGGAGGGAGAAGATGAAAGGACCAATAATAATG |
|  | B cat | GCCAGGCTAAGGGTCAAAATGGTATGCGTTT |
|  | NdeI 1 cat | GGGCCACATATGCCCAGAAG |
|  | E cat | TTATTGGTCCTTTCATCTTCTCCCTCCTTTC |
|  | F cat | TACCATTTTGACCCTTAGCCTGGCCCGTAG |
|  | 4.3 cat ecoR1 | CCCAAGCCCGAATTCTTTCCC |
|  | 5 cat | ACCCAGGTGTCCTCGAGG |
|  | 6 cat | CTGGACCGGGTCTACCCC |
| ∆*pcat*  *hygB* | NdeI 1 cat | GGGCCACATATGCCCAGAAG |
|  | E cat hygB KO rev | AGGCTTTTTCATCTTCTCCCTCCTTTCG |
|  | B cat hygB KO rev | AGGCTAAGGGCTATTCCTTTGCCCTC |
|  | A cat hygB KO for | GAGGGAGAAGATGAAAAAGCCTGAACTCA |
|  | 4.3 cat eco R1 | CCCAAGCCCGAATTCTTTCCC |
|  | F cat hygB KO for | AAAGGAATAGCCCTTAGCCTGGCCC |
| ∆*sod*  *hygB* | 1 fum ecoRi for | TCGCGGGAATTCAGGGGAAC |
|  | E sod hygB rev | TTCAGGCTTTTTCATACTTCACCTCCGCTTC |
|  | A sod hygB for | CGGAGGTGAAGTATGAAAAAGCCTGAACTCAC |
|  | B sod hygB rev | GCTATGGGGACTATTCCTTTGCCCTCG |
|  | F sod hygB for | GCAAAGGAATAGTCCCCATAGCCTTATAGCC |
|  | 4 SOD HygB rev hindIII | GCCTGAAGCTTGCGGTGG |
| ∆*nfo* | p1 ecoR1 nfo | GGCCTGGTGGAATTCCGCAAC |
|  | E nfo Htk rev | TGGTCCTTTCATCCCCCGAAGCCTACCACAGG |
|  | B nfo HTk | GGGCGCTCAAAATGGTATGCGTTTTG |
|  | A nfo htk | TGGTAGGCTTCGGGGGATGAAAGGACCAATAATAATG |
|  | p4 bamhI nfo | CCGGGATCCTGGTGAACCTG |
|  | F nfo for | ACGCATACCATTTTGAGCGCCCCACCC |
|  | p5 nfo | CCGTCCTCGTCTACCTCCTG |
|  | p6 nfo | GGAGGATAGATGGGCACGG |
| *rrsB:*:*pcat* | 1.2 ecori Hp 16S for | GTCCGGGGGGAATTCGAGGAGC |
|  | E cat::16S | GAATAAGCCAGGATTTCAAGATGGGGGCATGGACCTCC |
|  | G cat::16S for | ATGCCCCCATCTTGAAATCCTGGCTTATTCTAGCGCC |
|  | H cat::16S rev | AGGCTTTTTCATTTACTTGGCCTTCTCG |
|  | A hygB cat::16S for | GCCAAGTAAATGAAAAAGCCTGAACTCACCG |
|  | B hygB cat::16S rev | TCGAGGAAGTCCATCTATTCCTTTGCCCTCGG |
|  | F cat::16S rev | GCAAAGGAATAGATGGACTTCCTCGAGGCCCTTTC |
|  | 4 HindIII 16S rev | CTGCGAAAAGAAGCTTCTCCC |
| *rrsB:*:*sod* | 1.2 ecori Hp 16S for | GTCCGGGGGGAATTCGAGGAGC |
|  | E SOD::16S rev | CTTGCCGCTCAAGATGGGGGCATGG |
|  | G SOD::16S rev | CCCCATCTTGAGCGGCAAGGGGCTTTGTGAGG |
|  | H SOD::16S rev | CTTTTTCATTCAGGCCTTCTTGAAGAACTCC |
|  | A hygB SOD::16S for | CAAGAAGGCCTGAATGAAAAAGCCTGAACTCACCG |
|  | B hygB sod::16S rev | TCGAGGAAGTCCATCTATTCCTTTGCCCTCGG |
|  | F sod::16S rev | GCAAAGGAATAGATGGACTTCCTCGAGGCCCTTTC |
|  | 4 HindIII 16S rev | CTGCGAAAAGAAGCTTCTCCC |

^1^Underlined sequences indicate restriction enzyme cutting sites.
